# Non-diffractive, toric, extended depth-of-focus intraocular lenses in eyes with low corneal astigmatism

**DOI:** 10.1186/s40662-024-00380-7

**Published:** 2024-04-01

**Authors:** Francisco Pastor-Pascual, Paz Orts-Vila, Pedro Tañá-Sanz, Santiago Tañá-Sanz, Ramón Ruiz-Mesa, Pedro Tañá-Rivero

**Affiliations:** 1 Oftalvist, C/ Ruzafa 19, 46004 Valencia, Spain; 2 Oftalvist, Alicante, Spain; 3 Oftalvist, Jerez de la Frontera, Spain

**Keywords:** EDOF, Toric, Intraocular lens, Astigmatism, Cataract

## Abstract

**Background:**

To assess clinical outcomes after implanting toric, extended-depth-of-focus intraocular lenses (IOLs) to correct low corneal astigmatism in eyes with cataracts.

**Methods:**

47 eyes were implanted with the AcrySof IQ Vivity Toric DFT215 IOL. Main outcome measures were refractive error, monocular uncorrected and corrected distance (UDVA/CDVA), uncorrected and distance-corrected intermediate (UIVA/DCIVA), and uncorrected near and distance-corrected near (UNVA/DCNVA) visual acuities, monocular defocus curve, rotational stability, and IOLSAT and QUVID questionnaires. Patients were assessed at 3 months postsurgery.

**Results:**

All eyes had a postoperative spherical equivalent (SE) within ± 0.50 D and 97.87% (n = 46) had a refractive cylinder ≤ 0.50 D. The mean SE and refractive cylinder were − 0.10 ± 0.17 D and − 0.16 ± 0.24 D, respectively. The CDVA was ≥ 20/25 and ≥ 20/32 in 95.74% (n = 45) and 97.87% (n = 46) of eyes, respectively. The DCIVA was ≥ 20/32 in 85.11% (n = 40) of eyes and the DCNVA was ≥ 20/40 in 74.47% (n = 35). The mean values of CDVA, DCIVA, and DCNVA were − 0.02 ± 0.08, 0.14 ± 0.09, and 0.23 ± 0.12 logMAR, respectively. The defocus curve revealed good visual acuity at far and intermediate distances with a depth-of-focus of about 1.75 D. IOL rotation was 0.74 ± 1.13 degrees and all eyes had a rotation of less than 5 degrees. Patients reported either good or very good postoperative vision without eyeglasses under bright-light-conditions at distance (87.80%, 36/41) and intermediate distance (92.68%, 38/41). Between about 63.83%–72.34% (30–34) of patients reported no starburst, halos, or glare, or if experienced, were not bothersome.

**Conclusions:**

The Vivity toric IOL implanted in eyes with low-astigmatism provides accurate refractive outcomes, good visual acuity at different distances and excellent rotational stability.

*Trial Registration* The study was registered with the German Clinical Trials Register (DRKS00030579)

## Background

Different intraocular lens (IOL) designs based on refractive, diffractive, or a combination of both technologies have been developed to achieve good vision at different distances, from far to near vision, in patients with cataracts. A recent systematic review and meta-analysis of randomized controlled trials concluded that patients receiving multifocal IOL implants are more likely to be spectacle free but have a higher risk of certain visual phenomena and reduced contrast sensitivity [1]. The development of new designs based on the concept of increasing the depth of focus, i.e., extended depth-of-focus (EDOF) IOLs, have appeared on the market to help reduce spectacle dependence and improve the vision quality at intermediate distances.

The AcrySof IQ Vivity EDOF IOL (Alcon Labs, Fort Worth, TX, USA) was recently introduced and uses non-diffractive wavefront-shaping technology comprising two smooth surface transition elements working synergically. Good postoperative outcomes have been reported with this lens after cataract surgery [2, 3], but only a few peer-reviewed publications have focused specifically on the toric model of the lens [4–8]. Two studies show that about 60% of eyes undergoing cataract surgery have an astigmatism of up to 1.00 D (59.9% [9] and 58.7% [10]). Removing this astigmatism may further improve visual acuity after cataract surgery and implanting an EDOF IOL. If we consider that an astigmatism of about 0.50 D is roughly equivalent to 0.25 D of sphere producing a change in high-contrast visual acuity of approximately one logMAR line [11], although the effect of residual astigmatism may be somewhat greater [12]. As such, calculations for EDOF IOLs should consider a correction for the astigmatism to ensure patients obtain the best visual acuity.

To the best of our knowledge, no peer-reviewed publications have specifically studied the visual and refractive outcomes obtained with AcrySof IQ Vivity toric IOLs in eyes with low corneal astigmatism. Therefore, the aim of this clinical study was to assess the refractive and visual outcomes in a series of eyes with low astigmatism implanted with AcrySof IQ Vivity toric EDOF DFT215 IOLs.

## Methods

This prospective, observational study was approved by the Ethics Committee of the Hospital Clínico San Carlos (Madrid, Spain) and the Valencia Regional Committee of Observational Postmarketing Studies, CAEPRO (Valencia, Spain) (No. 22/304-O_P). All recruited patients provided written informed consent before they were enrolled, and the study was registered with the German Clinical Trials Register (DRKS00030579).

### Patients

We prospectively examined patients at two Oftalvist Clinics in Valencia and Alicante, Spain, between September 2022 and July 2023. The inclusion criteria were candidates for age-related cataract surgery with a corneal astigmatism ≤ 1.00 D (candidate for an EDOF DFT215 in at least one eye) and aged 55 to 85 years. The exclusion criteria were dry eye or tear film alteration assessed with a Keratograph 5M corneal topographer, glaucoma, retinal or corneal diseases, and previous corneal or intraocular surgery.

### Intraocular lens and surgical procedure

All eyes were implanted with AcrySof IQ Vivity toric EDOF DFT215 IOLs (Alcon Labs, Fort Worth, TX, USA) by various surgeons (FPP, POV, PTR). The DFT215 is a non-diffractive, UV-absorbing and blue-light-filtering EDOF lens made from a hydrophobic acrylic material with a refractive index of 1.55. The lens has a biconvex, aspheric optic with patented X-WAVE™ technology and a negative spherical aberration on the anterior surface, while the posterior surface is biconic, creating a toricity to correct the astigmatism. The profile of the lens has been analyzed in vitro [13]. It has an optic diameter of 6.0 mm, and the overall diameter is 13.0 mm. It also features Stableforce modified-L haptics with a haptic angle of 0 degree. The lenses are indicated for powers from + 10.00 to + 30.00 D in 0.50 D increments and available in IOL cylinder powers of 1.00 (DFT215), 1.50 (DFT315), 2.25 (DFT415), 3.00 (DFT515), and 3.75 D (DFT615). However, this study only used the DFT215 model.

Standard phacoemulsification cataract surgery was performed through a 2.2 mm, clear, temporal corneal incision using a topical anesthetic and the Centurion^®^ vision system (Alcon Labs, Fort Worth, TX, USA). We carried out a 5.0 mm diameter circular capsulorhexis and after removing the cataract and polishing the posterior capsule, the capsular bag was filled with 1.0% sodium hyaluronate (ProVisc from Alcon Labs, Fort Worth, TX, USA).

### Pre and postoperative assessment

All patients received a complete eye examination before and after the cataract surgery. The following measurements were made before surgery: logMAR monocular uncorrected distance visual acuity (UDVA) and corrected distance visual acuity (CDVA), subjective standard manual refraction and corneal topography with a Keratograph 5M topographer (Oculus Optikgeräte GmbH, Wetzlar, Germany). Other tests were also performed to assess ocular health including biomicroscopy, Goldmann applanation tonometry, and fundoscopy after pupil dilation. Swept-source optical biometry was performed using an IOLMaster 700 biometer (Carl Zeiss Meditec AG, Jena, Germany) and the IOL power calculation was performed with the Alcon online toric IOL calculator (Barrett Universal II formula). The target refraction in all cases was emmetropia. Only one eye in each patient was considered for the analysis. All patients were implanted bilaterally with AcrySof IQ Vivity IOLs and at least one eye with the DFT215 IOL model (T2). If both eyes were implanted with the T2 model, and therefore eligible considering the inclusion/exclusion criteria, the eye included in the analysis was selected randomly by a computer program.

Follow-up visits were carried out at 1 and 3 months postsurgery. At each visit, we assessed monocular logMAR UDVA, CDVA, uncorrected and distance-corrected intermediate visual acuity (UIVA and DCIVA) at 66 cm, and uncorrected and distance-corrected near visual acuity (UNVA and DCNVA) at 40 cm using ETDRS charts. We also measured the monocular defocus curve, from + 1.00 to − 3.00 D (0.50 D increments), to assess the useful range of vision. Refraction parameters [sphere, cylinder, and manifest refraction spherical equivalent (MRSE)] were measured, and an astigmatism vector analysis performed with the double-angle tool [14]. IOL rotation stability was assessed at each visit. The procedure was recorded on video and a suitable screenshot selected at the end of the surgery using the ORA System® (Alcon Labs, Fort Worth, TX, USA). A line between the two episcleral landmarks (always the same landmarks) was used as a reference for all examinations. The meridional position of the IOL was defined as the angle between the reference line and the IOL axis lines. IOL rotation between consecutive follow-up visits was determined by measuring the change in angle [15]. In the postoperative follow-up visits, photographs were taken during the slit-lamp examination (after pupil dilation). Rotational stability was assessed at 1 and 3 months postsurgery, using the end of surgery photograph as the baseline measurement. Two validated questionnaires were administered before the intervention and at the last follow-up visit: one questionnaire was about patient satisfaction regarding their vision (IOLSAT) and another concerning the quality of their vision (QUVID) [16]. Both questionnaires, proprietary of Alcon, ask patients to score their satisfaction with their vision at different distances and the frequency and severity of any visual disturbances. Any surgical complications or postoperative adverse events were also recorded. The analysis of the outcomes was done for the 3-month postsurgery data.

### Statistical analysis and sample size calculation

The statistical analysis was carried out using SPSS software (version 22.0, IBM Corp., Armonk, New York, USA). All values are presented as mean ± standard deviation (SD). Assuming a sample size of 45 eyes from 45 patients, a 95% confidence interval, and a SD of 0.12 logMAR distance visual acuity (based on a similar paper [17]), then the primary estimate will have a precision of 0.0335 logMAR, which we considered to be sufficient for this study.

## Results

This study assessed 47 eyes of 47 patients implanted with AcrySof IQ Vivity toric IOLs, T2 model. The patient demographics and preoperative ocular measurements are summarized in Table 1. No complications or adverse events were reported either during surgery or up to the final follow-up visit.

**Table 1:** Demographics and preoperative measurements of participants shown as mean, standard deviation (SD), and range

Parameter	Mean ± SD (range)
Eyes (n)	47
Age (years)	68.04 ± 7.22 (55 to 81)
Sphere (D)	0.02 ± 3.27 (− 15.00 to 7.00)
Refractive cylinder (D)	− 0.74 ± 0.54 (− 2.00 to 0.00)
Spherical equivalent (D)	− 0.28 ± 3.33 (− 15.75 to 6.75)
CDVA (logMAR)	0.13 ± 0.14 (0.60 to 0.00)
K1 (D)	43.53 ± 1.48 (39.86 to 46.78)
K2 (D)	44.22 ± 1.43 (40.91 to 47.61)
Axial length (mm)	23.71 ± 1.18 (20.99 to 26.83)
ACD (mm)	3.10 ± 0.33 (2.44 to 4.07)
IOL spherical power (D)	20.65 ± 3.41 (10.50 to 28.50)

*CDVA* = corrected distance visual acuity; *K* = keratometry; *ACD* = anterior chamber depth; *IOL* = intraocular lens; *SD* = standard deviation

All lenses had a cylindrical power of 1.00 D (DFT215)

**Fig. 1:**
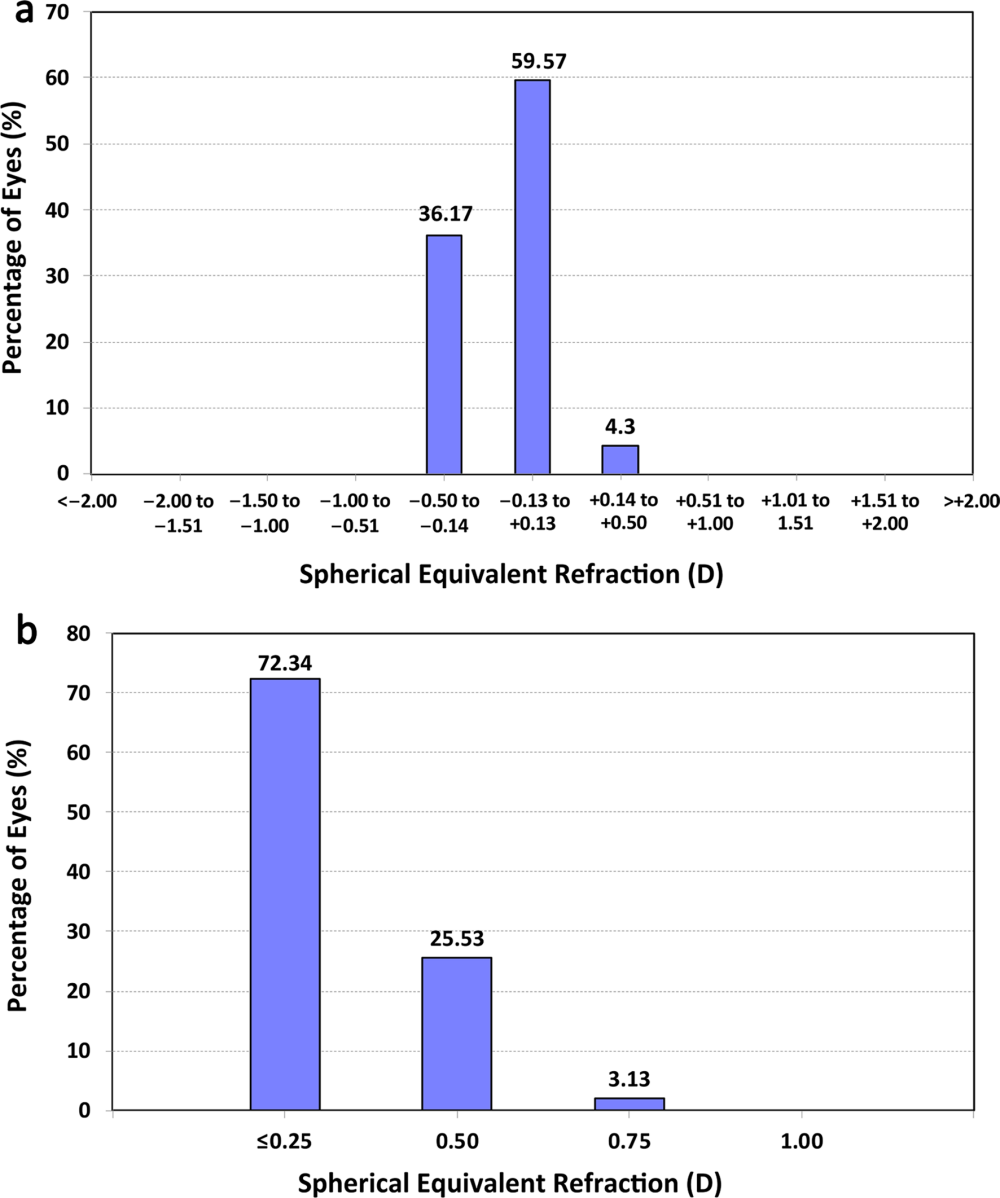
Distribution of spherical equivalent refraction (**a**) and refractive cylinder (**b**) at 3 months postsurgery

**Fig. 2:**
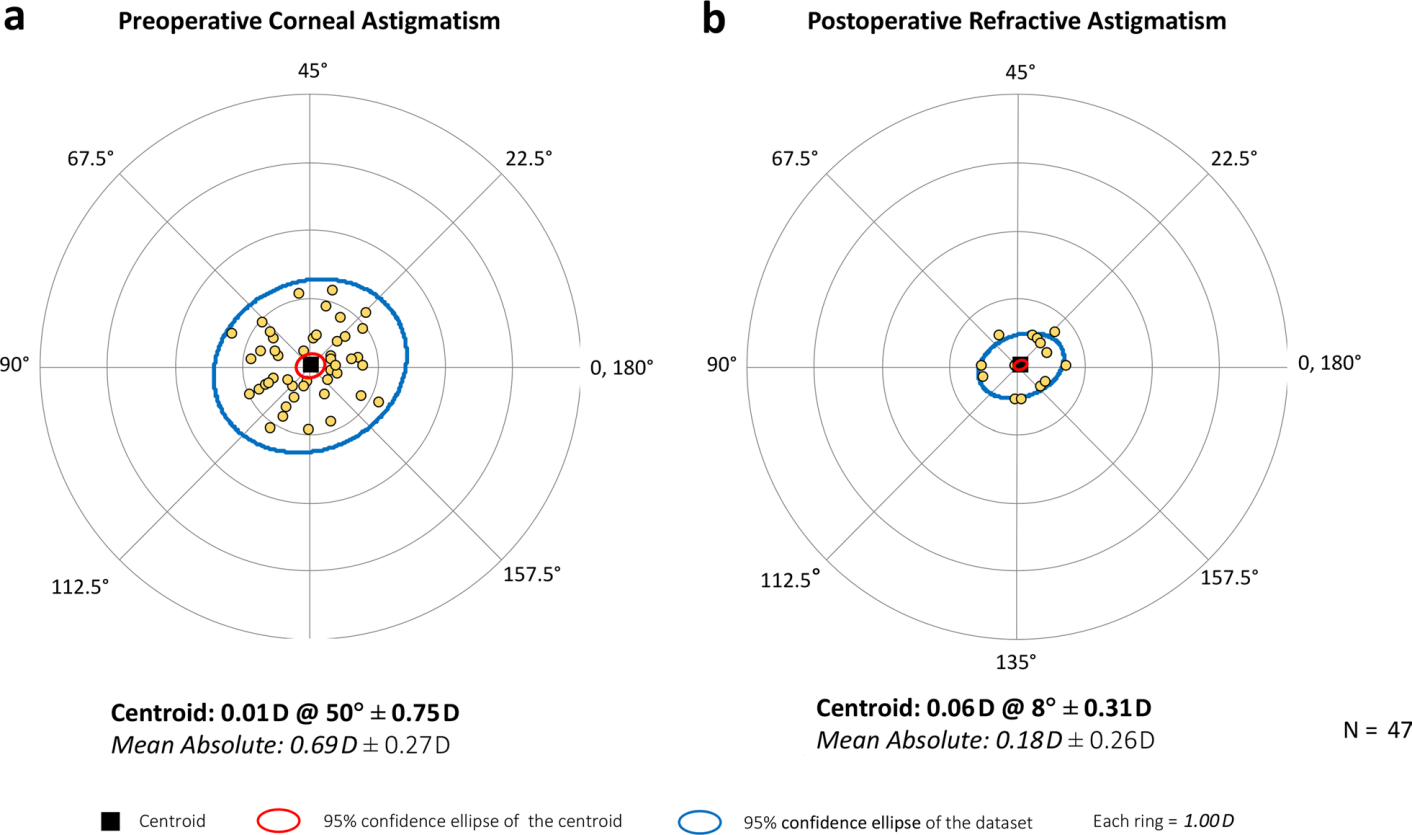
Double-angle plots for preoperative corneal astigmatism (**a**) and postoperative refractive astigmatism (**b**) (3 months postsurgery) applying the double-angle tool [14]. Centroids, mean absolute values with standard deviations, and 95% confidence ellipses of the centroid and dataset are also shown

Figure 1a shows the distribution of spherical equivalent (SE) postsurgery. The graph shows that 59.57% of eyes (28) were within ± 0.13 D and 36.17% (17) in the range − 0.14 to − 0.50 D. All implanted eyes were within ± 0.50 D. The mean postoperative SE was − 0.10 ± 0.17 D (range: − 0.50 to 0.25 D). The analysis of the postoperative refractive cylinder (Fig. 1b) revealed that 72.34% (34) of eyes were ≤ 0.25 D and 97.87% (46) were ≤ 0.50 D. Specifically, the mean postoperative refractive cylinder was − 0.16 ± 0.24 D (0.00 to − 0.75 D). Double-angle plots of preoperative corneal astigmatism (Fig. 2a) and postoperative refractive astigmatism (right) showed a concentration of results at the origin (0, 0), which corresponds to eyes free of astigmatism. The preoperative mean absolute corneal astigmatism was 0.69 ± 0.27 D and the postoperative mean absolute refractive astigmatism was 0.18 ± 0.26 D.

**Fig. 3:**
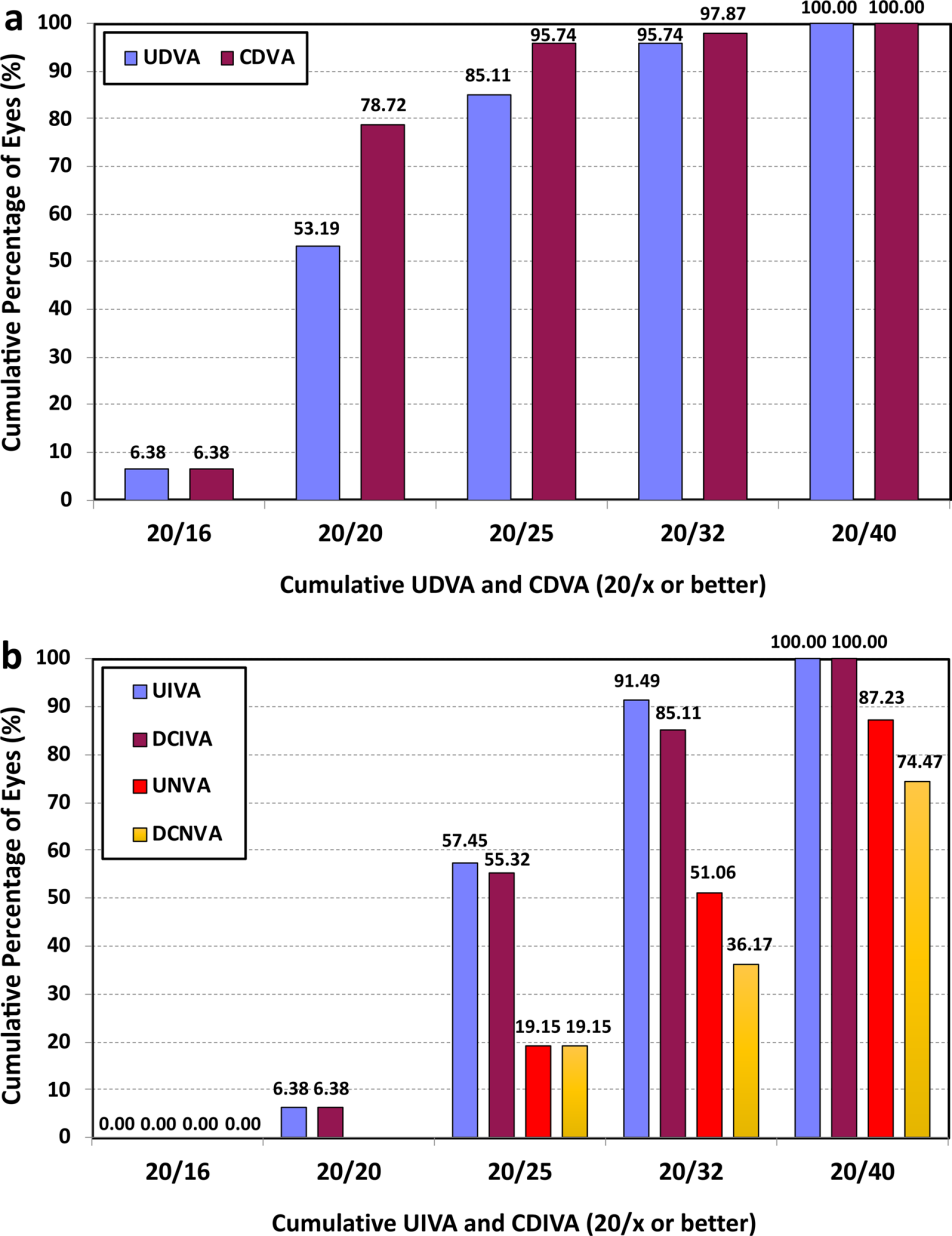
Cumulative percentage of eyes at 3 months postsurgery with different degrees of uncorrected and corrected distance visual acuity (UDVA and CDVA) (**a**), and uncorrected and distance-corrected intermediate visual acuity at 66 cm (UIVA and DCIVA) and uncorrected and distance-corrected near visual acuity at 40 cm (UNVA and DCNVA) (**b**)

**Fig. 4:**
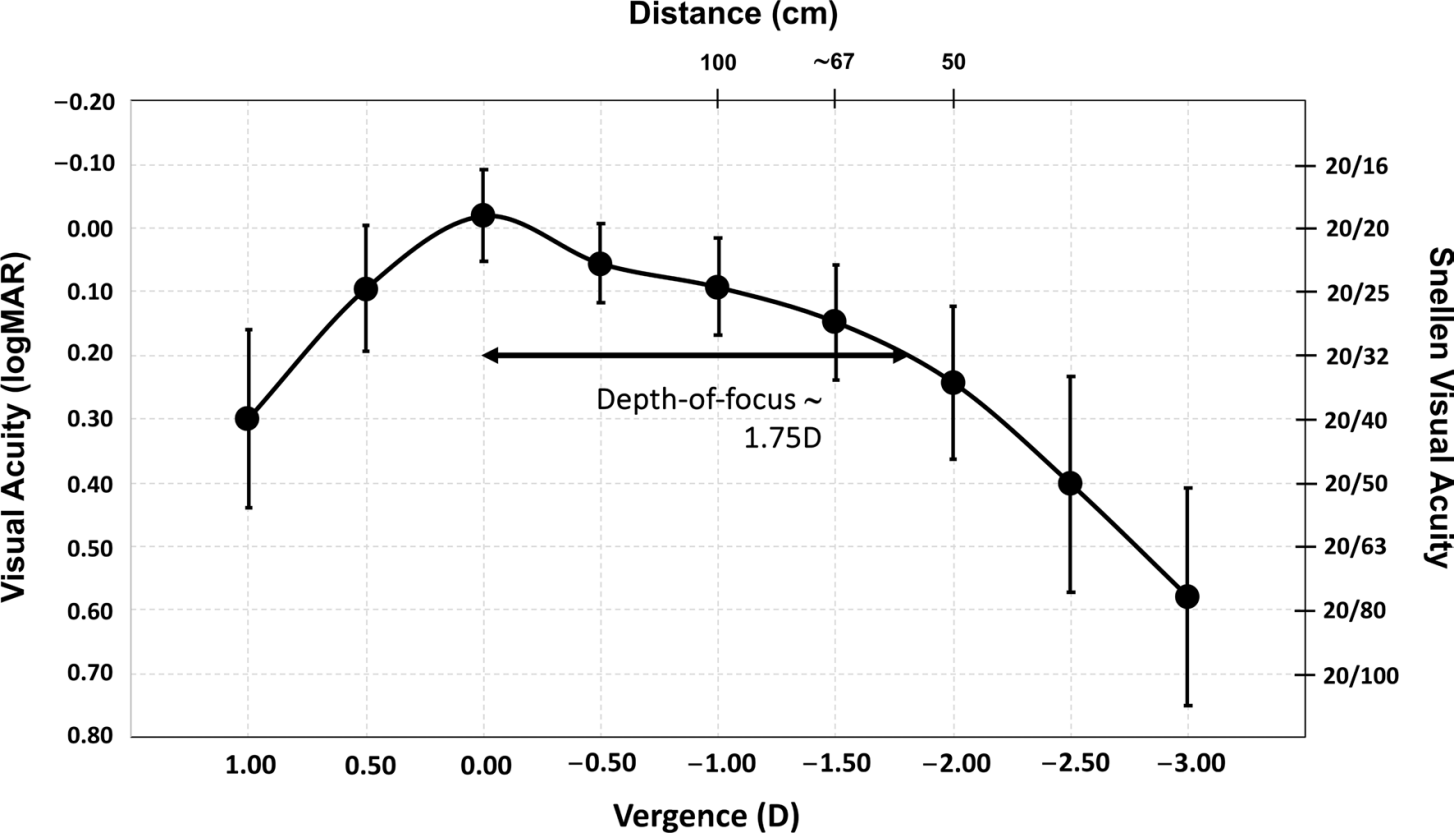
Mean monocular logMAR visual acuity with best correction for distance based on the vergence chart for AcrySof IQ T2 Vivity IOLs at 3 months postsurgery. Error bars are the standard deviation. Right y-axis shows the Snellen visual acuity in feet and top x-axis shows the distance (cm). Depth-of-focus was defined as the range of lens powers that achieved a mean acuity of 20/32 or better (from 0.00 D of vergence)

Regarding the visual acuity results, Fig. 3 provides the cumulative percentage of eyes that achieved given monocular UDVA and CDVA values (top), and UIVA, DCIVA, UNVA, and DCNVA scores (bottom) at 3 months postsurgery. The CDVA was 20/25 or better in 95.74% (45) of eyes and 20/32 or better in 97.87% (46) of eyes. The DCIVA was 20/25 or better in 55.32% (26) of eyes and 20/32 or better in 85.11% (40) of eyes, while the DCNVA was 20/32 or better in 36.17% (17) and 20/40 or better in 74.47% (35) of eyes. The mean values of postoperative monocular UDVA, UIVA, and UNVA were 0.02 ± 0.09, 0.12 ± 0.07, and 0.21 ± 0.11 logMAR, respectively. The mean values of postoperative monocular CDVA, DCIVA, and DCNVA were − 0.02 ± 0.08, 0.14 ± 0.09, and 0.23 ± 0.12 logMAR, respectively. Figure 4 shows the mean monocular logMAR visual acuity with best correction for distance from 1.00 to − 3.00 D. There is a peak in visual acuity for distance vision (vergence of 0.00 D), followed by a steady reduction as the negative vergence increases in magnitude (intermediate and near vision). The depth-of-focus was defined as the range of lens powers that achieved a mean acuity of 20/32 or better (from 0.00 D of vergence), and for our results it spanned about 1.75 D.

**Fig. 5:**
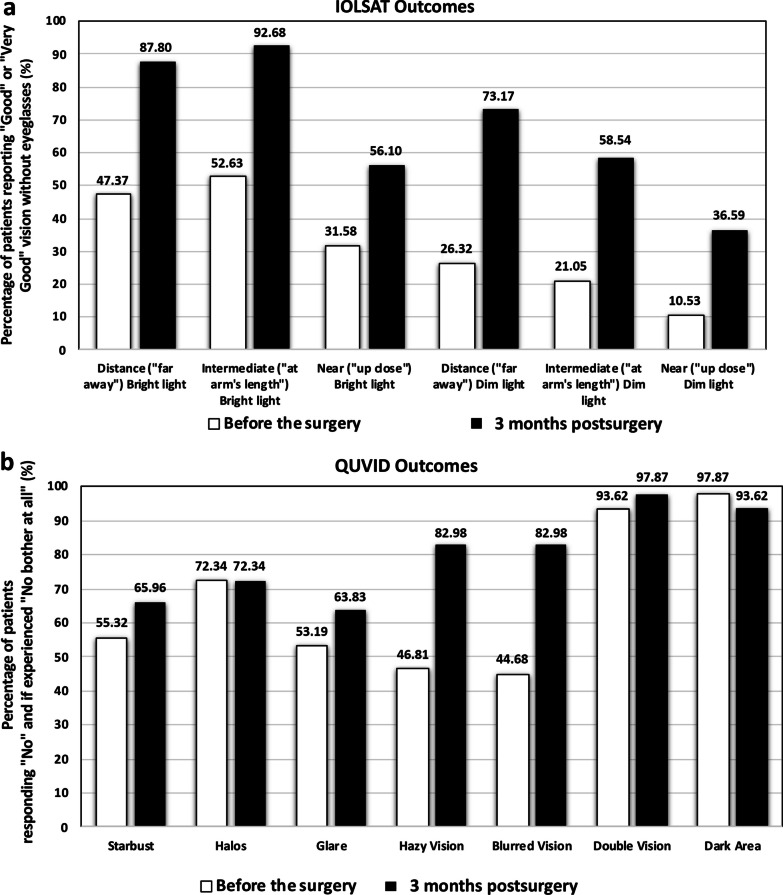
Percentage of patients, before surgery and at 3 months postimplant, reporting “good” or “very good” vision without eyeglasses at different distances under bright and dim light conditions (IOLSAT questionnaire, **a**) and percentage who responded “no” or if experienced, “not bothered at all” for certain visual disturbances (QUVID questionnaire, **b**). Note that 100% represents the best outcomes, that is, no patients reporting postoperative issues with that visual disturbance

At 3 months, the lens had a mean rotational stability of 0.74 ± 1.13 degrees (range: 0 to 4 degrees). Note that no significant rotation was reported in any of the eyes during follow-up; all eyes had a rotation of less than 5 degrees. Analysis of the IOLSAT and QUVID questionnaires revealed that patients were satisfied with their vision. Figure 5a shows the percentage of patients who reported “good” or “very good” vision without eyeglasses at different distances under bright and dim light conditions (IOLSAT questionnaire) before surgery and at 3 months postimplant. The rate of patients who reported good/very good postoperative vision without eyeglasses under bright light conditions was 87.80% (36/41 patients) at distance and 92.68% (38/41 patients) for intermediate. The number of patients who perceived good/very good vision approximately doubled between the pre- and postoperative questionnaires for all distances. The pre- and postoperative difference was even greater under dim light conditions. Figure 5b shows the proportion of patients reporting “no” or “not bothered at all” if they were unaffected or untroubled by certain visual disturbances (QUVID questionnaire) before surgery and at 3 months postimplant. Between about 63.83%–72.34% (30–34) of patients reported no starburst, halos, or glare after cataract surgery, or if they experienced them, did not find them bothersome. This is about 10% higher than before the operation. The percentages for four other visual disturbances at 3 months were over 80% (≥ 39 patients).

## Discussion

Previous clinical and in vitro studies have analyzed the visual, optical, and functional quality of life outcomes obtained with different commercially available EDOF IOLs [4–6]. They put forward that these lenses can improve intermediate vision while providing a good level of far vision. However, there is a wide variety of EDOF lenses with different optical designs and their resulting impact on the patient’s visual performance can vary significantly. Previous studies have reported on the outcomes achieved with AcrySof IQ Vivity non-toric [2, 3] and toric [4–8] models. However, to the best of our knowledge, no clinical studies have published any results for the toric model implanted in eyes with low corneal astigmatism. Therefore, the present study aimed to fill this gap.

Our results revealed that the lens offers excellent refractive accuracy in both spherical and astigmatism correction (SE and refractive astigmatism were both less than one quarter of a diopter: − 0.10 ± 0.17 D and − 0.16 ± 0.24 D, respectively). This is also supported by the concentration of dots for postoperative refractive astigmatism around the origin in Fig. 2 (mean absolute of 0.18 ± 0.26 D). Our results show that the lens provides an extended range of vision, as evidenced by the monocular defocus curve in Fig. 4, with a peak in visual acuity for distance vision (vergence of 0.00 D) and a continuous reduction for intermediate and near vision (down to − 3.00 D). This gave a depth-of-focus of approximately 1.75 D. We believe better results can be expected if a binocular assessment is performed. The CDVA was ≥ 20/25 in 95.74% of eyes and ≥ 20/32 in 97.87%, and we found a DCIVA ≥ 20/32 in 85.11% and  ≥ 20/40 in 74.47% of eyes. We also assessed the rotational stability of the lens as it is important for any platform aiming to correct astigmatism. The rotation was less than 5 degrees in all eyes and the mean absolute rotation was 0.74 ± 1.13 degrees. According to ISO11979-7:2024, the absolute value of rotation should be less than 10 degrees in 90% of implanted eyes [18]. However, we consider this amount of rotation to be clinically insignificant and, based on our values, we conclude that the lens had a high degree of rotational stability when implanted in the capsular bag. This stability correlates with effective postoperative astigmatism correction (mean refractive cylinder of − 0.18 ± 0.26 D) and hence good unaided distance visual acuity (85.11% of patients had a monocular UDVA of 20/25 or better, Fig. 3a). The rotational stability of the AcrySof IQ toric IOL, the platform used for the Vivity IOL, has been reported in previous studies with mean absolute rotations ranging from 2.20 ± 2.20 to 4.24 ± 4.10 degrees and with at least 88% of lens rotating less than 10 degrees [19–21]. An undesired rotation of a toric IOL by 5 degrees translates into a theoretical loss of approximately 17% of the astigmatic effect [22, 23]. This corresponds to 0.17 D of lost astigmatism correction for a T2 model although the baseline residual astigmatism and lost astigmatism correction do not generally share a common axis and do not add linearly [7]. Our results also show that the AcrySof IQ Vivity IOL confers good vision without eyeglasses (see the high percentage of patients reporting “good” or “very good” vision under bright light at far and intermediate distances in Fig. 5a). The lens proved to be safe in terms of visual disturbances as shown by the good results for seven types of disturbance (see Fig. 5b). This correlates with the fewer optical phenomena reported previously with the non-toric model, which are both significantly less than those observed with other EDOF or multifocal IOLs [24]. Our results were similar to those reported by McCabe et al. [3] with the non-toric model at 6 months postsurgery.

As explained in the introduction, some studies have published data on patients implanted with either non-toric or toric AcrySof IQ Vivity IOLs [4–6], but they did not include separate sub-analyses for the toric model for a proper assessment of its performance. In a non-interventional study, Gundersen and Potvin [4] assessed the effect of spectacle-induced low myopia (− 0.50 D and − 1.00 D) in the non-dominant eye on the binocular defocus curve in a sample of 40 patients implanted with AcrySof IQ Vivity IOLs. As mentioned, the study considered both non-toric and toric lenses and the outcomes were reported as a single sample, so we cannot analyze any specific results for the toric model. In a similar study, Arrigo et al. [5] reported on real-life experiences in 108 eyes implanted with either non-toric or toric models of the AcrySof IQ Vivity IOLs. They analyzed the visual acuity and refractive outcomes and administered a quality of vision questionnaire. They decided not to perform dedicated analyses on these eyes because of the relatively low number of toric IOLs implanted although the overall patient feedback was very good and the authors concluded that the lens is a well-tolerated choice to correct far and intermediate vision with very few postoperative complications or visual symptoms. Finally, Kandavel et al. [6] also examined both toric (n = 29) and non-toric (n = 35) models of the AcrySof IQ Vivity IOLs. They concluded that their findings supported previous studies in that both toric and non-toric models achieve a similar visual disturbance profile to monofocal IOLs while offering improved near and intermediate vision with less dependence on spectacles.

Only two recent studies have specifically assessed the AcrySof IQ Vivity toric IOL [7, 8]. Barber et al. [7] studied the toric model in 35 eyes of 35 patients. They analyzed the outcomes obtained at 1 month postimplant with the T3 (74%), T4 (17%), and T5 (8.6%) models; however, unfortunately, no eyes were implanted with the T2 model. The authors used the Barrett Universal II toric formula for the IOL power calculation and the target refraction was emmetropia. They also used the ORA SYSTEM® to guide the selection of toric IOL power and alignment. The mean absolute postoperative IOL rotation at 1 month was 1.1 ± 0.2 degrees and remained stable throughout the postoperative follow-up period (1 day and 1 week, *P* = 0.58). They found that the maximum postoperative rotation observed at 1 month was 3 degrees. Our mean value was 0.74 ± 1.13 degrees, ranging from 0 to 4 degrees, which is in tandem with their outcomes. They measured IOL orientation with digital photography using a slit lamp mounted on an iPhone (Apple; Cupertino, California, USA) and a toric reticle from the toriCAM app (Graham Barrett; version 4.0) as a reference mark [25]. The resulting photographs were analyzed to determine the IOL axis. They observed that the residual refractive astigmatism was ≤ 0.50 D in 94% of eyes and ≤ 1.00 D in 100%. At 97.87% and 100% of eyes for the same levels of residual refractive astigmatism, our results broadly agreed with theirs. At the 1 month follow-up, their mean residual regular astigmatism was 0.21 ± 0.047 D, which was also similar to ours (< 0.25 D). Barber et al. [7] also reported mean monocular UDVA and CDVA scores of 0.18 ± 0.022 and 0.078 ± 0.017 logMAR, respectively. At intermediate distances, their mean monocular UIVA and DCIVA scores were 0.27 ± 0.040 and 0.17 ± 0.025 logMAR, respectively, while these results were slightly better in our study: 0.12 ± 0.07 and 0.14 ± 0.09 logMAR. Finally, the same authors [7] concluded that Vivity toric IOLs had excellent postoperative rotational stability without any lenses rotating by more than 3 degrees at the final 1-month follow-up visit and was effective and predictable for astigmatism correction.

The more recent of the two clinical studies, published by Nguyen et al. [8], assessed 20 patients treated bilaterally with AcrySof IQ Vivity toric IOLs, implanting the T2 model in 10% of eyes, the T3 in 35%, the T4 in 32.5%, and the T5 in 22.5% (the mean IOL cylinder power was 2.01 ± 0.71 D for the whole sample). They used the Barrett Universal II formula/Barrett Toric Calculator (American Society of Cataract and Refractive Surgery) with a target refraction of emmetropia or first minus in all eyes. At 3 months, they reported a mean SE of − 0.55 ± 0.45 D and − 0.63 ± 0.48 D for automated and manual refraction, respectively. The mean residual astigmatism was 0.49 ± 0.34 D and 0.54 ± 0.37 D for automated and subjective refraction. Our results were better, as the mean values were closer to emmetropia. All eyes except one (2.5%) had a residual astigmatism ≤ 1.00 D, whereas in our study all eyes ≤ 1.00 D. At 3 months, they found the binocular UDVA, UIVA (66 cm), and UNVA (40 cm) were 0.01 ± 0.06, 0.08 ± 0.08, and 0.14 ± 0.07 logMAR, respectively, and the monocular CDVA, DCIVA (66 cm), and DCNVA (40 cm) were 0.02 ± 0.06, 0.08 ± 0.08, and 0.05 ± 0.08 logMAR. Our mean values were better and similar for distance vision but worse for intermediate and near: − 0.02 ± 0.08, 0.14 ± 0.09, and 0.23 ± 0.12 logMAR, respectively. IOL rotation (measured using a slit lamp and by rotating a thin beam of light until it aligned with the IOL axis reference mark) from the intended placement axis was 2.5 ± 1.7 degrees at 1 week and 1.7 ± 1.7 degrees at 3 months postimplant. None of the lenses deviated by more than 7 degrees. As previously discussed, our mean rotational value was low, and we found rotation only up to 4 degrees. They administered the QUVID questionnaire to all patients at the 3-month follow-up visit and to 75% of patients (15 of 20) before surgery. All 20 patients reported they were satisfied with their visual outcomes and desired no further surgery. The mean preoperative QUVID score was 4.1 ± 5.3 and it rose to 22.5 ± 18.0 at 3 months postimplant (*P* < 0.01). Half of the patients (50%) reported no postoperative visual symptoms, whereas the symptoms reported as present “most of the time” were halo (n = 1, 5%), glare (n = 2, 10%), and hazy vision (n = 1, 5%). No patients reported any symptoms that bothered them “quite a bit” or “very much”, and the severity of the visual disturbances was moderate at most for all patients, with hazy vision (n = 2, 10%) being the most common moderately severe symptom. Our results revealed that about 63.83 and 72.34% of patients reported no starburst, halos, or glare, or if they experienced them, did not find them bothersome. For the other four visual disturbances we evaluated, between 82.92% and 97.87% of patients were untroubled at 3 months postimplant.

Unfortunately, there is nothing in the literature about defocus curves for the Vivity toric model although we can compare our results with those obtained with the non-toric model. For example, Bala et al. [2] conducted an international clinical study with 156 patients implanted bilaterally with Vivity non-toric lenses. Based on the binocular defocus curve at 6 months, they determined that patients achieved ≤ 0.0 logMAR for visual acuities from + 0.50 to − 0.50 D, < 0.1 logMAR down to − 1.50 D, and < 0.2 logMAR down to − 2.00 D (50 cm). The defocus curve of ≤ 0.0 logMAR (20/20) obtained in the defocus range of + 0.50 to − 0.50 D suggests the non-toric lens is tolerant to low amounts of residual refractive error. In another study, McCabe et al. [3] assessed 107 patients at 6 months postimplant and found that the Vivity non-toric IOL provided an extended monocular depth of focus compared to the monofocal AcrySof IQ IOL (increase of 0.54 D at 0.2 logMAR). Our results (see Fig. 4) were slightly lower than those of McCabe et al. [3]. Note that our defocus curve was measured under monocular conditions so we can expect to see better results if measured under binocular conditions due to binocular summation [26].

Nevertheless, we obtained a large depth of focus, about 1.75 D, which gives patients a good continuous range from distance to intermediate vision. These two multicenter studies reported good refractive and visual acuity outcomes for the non-toric model at 6 months. Specifically, Bala et al. [2] found that 84.7% of patients achieved a mean absolute SE ≤ 0.50 D (mean of − 0.15 ± 0.32 D). The mean monocular CDVA, DCIVA, and DCNVA were − 0.008 ± 0.007, 0.161 ± 0.013, and 0.414 ± 0.013 logMAR, respectively. Meanwhile, McCabe et al. [3] reported that 91.6% of eyes achieved a SE within ± 0.50 D (mean 0.049 ± 0.345 D). The mean monocular CDVA, DCIVA, and DCNVA were 0.016 ± 0.009, 0.148 ± 0.012, and 0.359 logMAR. Our values were similar for refraction (100% of eyes with SE within ± 0.50 D, mean − 0.10 ± 0.17 D) and distance and intermediate vision, and better for near vision (− 0.02 ± 0.08, 0.14 ± 0.09, and 0.23 ± 0.12 logMAR, for CDVA, DCIVA, and DCNVA, respectively). Limitations of our study include reduced sample size and the inclusion of data of several surgeons in our cohort.

## Conclusion

Our results show that implanting AcrySof IQ Vivity T2 IOLs in cataract patients with low corneal astigmatism offers excellent visual performance at different distances with good refractive outcomes and rotational stability. Patients reported good outcomes, as assessed using vision satisfaction and quality of vision questionnaires. Future studies should consider looking at high toric models of the same lens to confirm these outcomes in eyes with high degrees of corneal astigmatism.

## Data Availability

All data generated or analyzed during this study are included in this published article.
